# Supplementary material for: Glutamatergic synaptic resilience to overexpressed human alpha-synuclein
Source: NPJ Parkinsons Dis. 2025 Aug 12;11:238. doi: 10.1038/s41531-025-01085-x (PMC12343942; doi:10.1038/s41531-025-01085-x)
Supplement: Supplementary file 1 — Supplementary Information [file 41531_2025_1085_MOESM1_ESM.pdf]

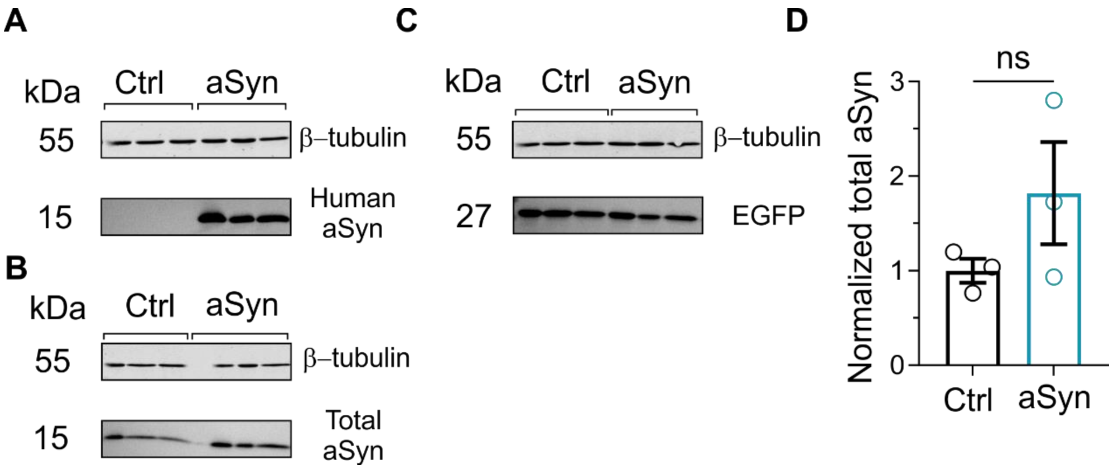

Supplementary Figure 1. Increased expression of aSyn at continental hippocampal mouse neurons infected with haSyn lentivirus.

Primary hippocampal continental cultures from mice were infected with lentivirus mediating the expression of either eGFP alone or haSyn and eGFP. Representative immunoblots of human aSyn (A), total aSyn (B), and eGFP (C) levels. (D) 1.82-fold increase in total aSyn levels in haSyn-infected neurons (n = 3 independent cultures). β-tubulin was used as a loading control. All data are expressed as mean ± SEM; Student's t-test.

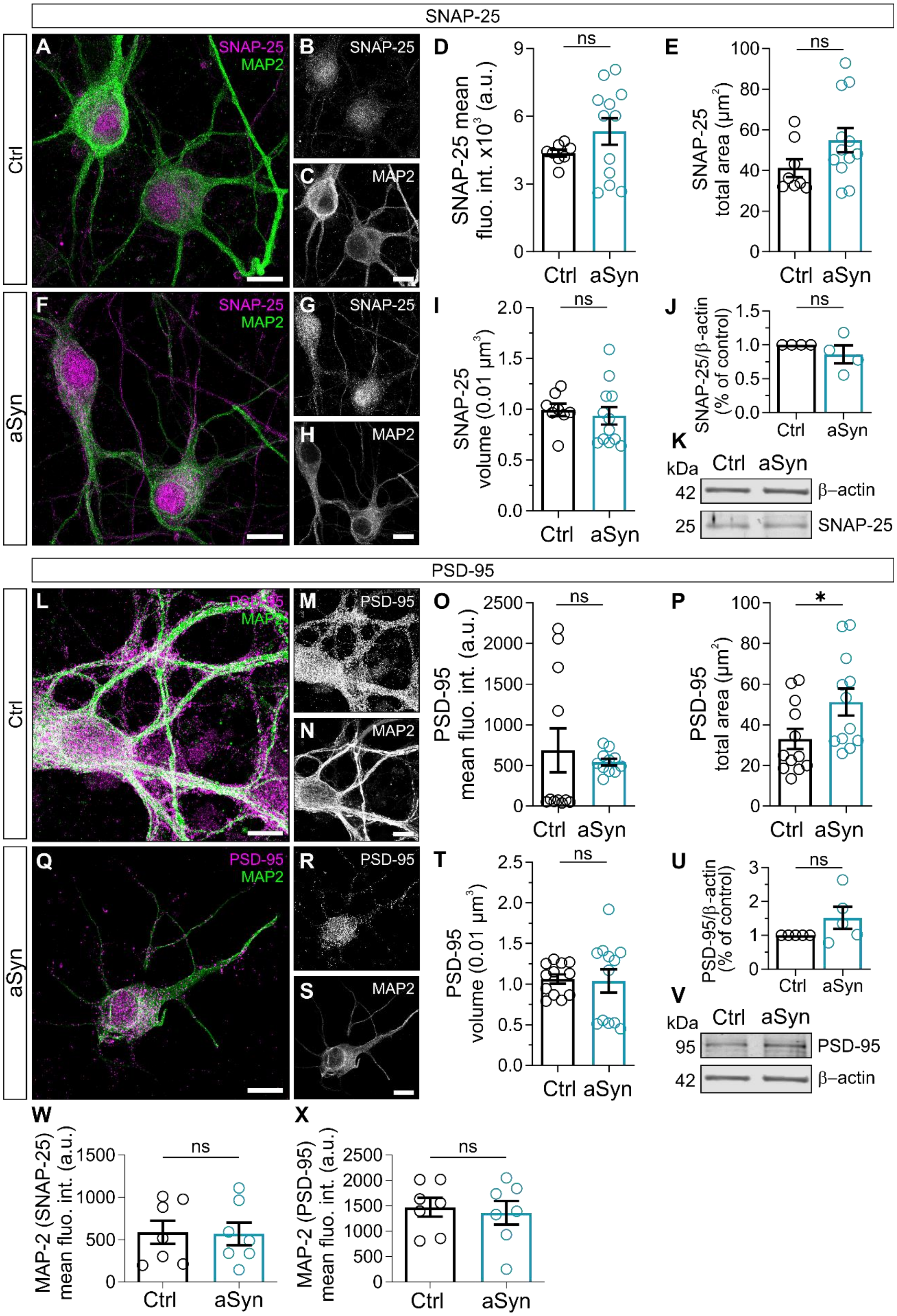

SNAP-25
Ctrl
aSyn
A
SNAP-25
MAP2
B
C
D
SNAP-25 mean fluo. int. x10³ (a.u.)
ns
E
SNAP-25 total area (μm²)
F
G
H
I
SNAP-25 volume (0.01 μm³)
J
SNAP-25/β-actin (% of control)
K
kDa
42
25
β–actin
PSD-95
L
M
N
O
PSD-95 mean fluo. int. (a.u.)
P
PSD-95 total area (μm²)
*
Q
R
S
T
PSD-95 volume (0.01 μm³)
U
PSD-95/β-actin (% of control)
V
95
W
MAP-2 (SNAP-25) mean fluo. int. (a.u.)
X
MAP-2 (PSD-95) mean fluo. int. (a.u.)

Supplementary Figure 2. Lentivirus-mediated overexpression of haSyn do not change the SNAP-25 and PSD-95 fluorescence

(A-H) Representative images of immunocytochemistry of continental hippocampal neurons infected with eGFP or haSyn lentivirus against SNAP-25 (B,G) and MAP2 (C,H). Overlays are shown (A,F). Scale bars = 10 μm. Quantification of SNAP-25 synaptic mean fluorescence intensity (D), synaptic area (E) and synaptic volume (I) (n = 8-12/2 neurons/independent cultures). (J-K) Representative immunoblots (K) and respective quantification (J) of SNAP-25 levels assessed in primary neurons transduced with lentivirus encoding for eGFP or haSyn for up to DIV21 (n = 4 independent cultures). β-actin was used as a loading control. (L-U) Representative images of immunocytochemistry of continental hippocampal neurons infected with eGFP or haSyn lentivirus against PSD-95 (M,R) and MAP2 (N,S). Overlays are shown (L,Q). Scale bars = 10 μm. Quantification of PSD-95 synaptic mean fluorescence intensity (O), synaptic area (P) and synaptic volume (T) (n = 12/2 neurons/independent cultures). (U-V) Representative immunoblots (V) and respective quantification (U) of PSD-95 levels assessed in primary neurons transduced with lentivirus encoding for eGFP or haSyn for up to DIV21 (n = 5 independent cultures). β-actin was used as a loading control. (W-X) Quantification of MAP-2 mean fluorescence intensity in continental hippocampal neurons infected with eGFP or haSyn lentivirus and co-stained with MAP2 and SNAP-25 (W), or PSD-95 (X). Data are expressed as mean ± SEM; Student's t-test; * p-value ≤ 0.05.

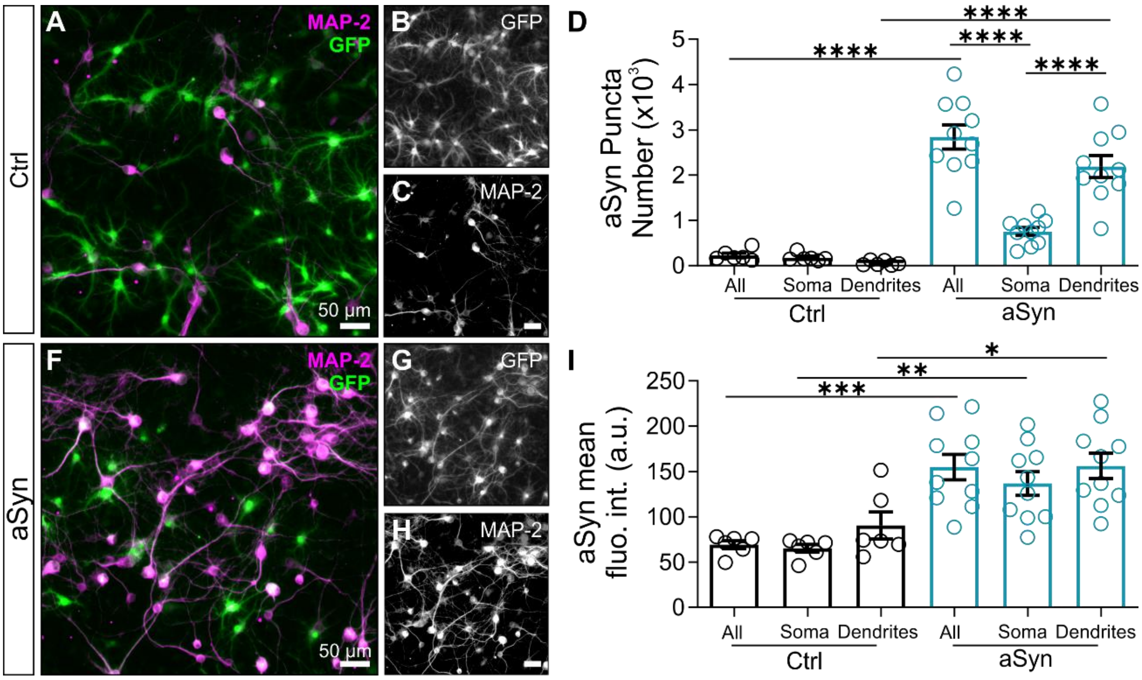

Supplementary Figure 3. Lentivirus-mediated overexpression of haSyn changes the distribution of aSyn-positive puncta in neurons

(A-H) Representative images of immunocytochemistry of continental hippocampal neurons infected with eGFP or haSyn lentivirus against GFP (B,G) and MAP2 (C,H). Overlays are shown (A,F). Scale bars = 50 µm. Quantification of aSyn-positive puncta (D) and aSyn mean fluorescence intensity (I) (n = 6-10/1 neurons/independent culture). All data are expressed as mean ± SEM; one-way ANOVA followed by Tukey's multiple comparisons test; * p-value ≤ 0.05; ** p-value ≤ 0.01; *** p-value ≤ 0.001; **** p-value ≤ 0.001.
